# *APOC3* Interference for Familial Chylomicronaemia Syndrome

**DOI:** 10.17925/EE.2022.18.2.82

**Published:** 2022-11-02

**Authors:** Robert A Hegele

**Affiliations:** Departments of Medicine, (Division of Endocrinology) and Biochemistry, Western University, London, Canada

**Keywords:** Apolipoprotein C-II, apolipoprotein C-III, chylomicrons, hyperlipoproteinemia type 1, hyperlipoproteinemia type 5, lipoprotein lipase, lipoproteins, pancreatitis, recessive disorder, triglycerides

## Abstract

Patients with familial chylomicronaemia syndrome (FCS) have severe hypertriglyceridaemia due to genetically absent lipolytic capacity. They have a poor response to conventional therapies. To reduce the risk of potentially fatal pancreatitis, the management of FCS relies principally on a strict low-fat diet, which is difficult to follow and compromises quality of life. Targeted reduction of apolipoprotein C-III using new anti-*APOC3* agents, such as the short interfering RNA ARO-APOC3, represents a promising approach to correct the severe biochemical disturbance in FCS.

Chylomicronaemia refers to the pathological presence of large, circulating, intestinally derived chylomicrons.^1^ Clinical features of chylomicronaemia syndrome include lipaemic plasma, lipaemia retinalis, eruptive xanthomas, hepatosplenomegaly, neurological symptoms such as mental fog and mood changes and, most importantly, increased risk of acute pancreatitis.^1,2^ When smaller atherogenic triglyceride (TG)-rich lipoproteins are concomitantly elevated, the risk of myocardial infarction and stroke is also increased.^2^ Fasting plasma TG concentration of >10 mmol/L (>880 mg/dL), which occurs in approximately 1 in 400 North American adults,^3^ is associated with chylomicronaemia. Chylomicrons accumulate when endothelial-bound lipoprotein lipase (LPL) becomes saturated by excessive levels of TG-rich lipoproteins.^1,2^ Secondary factors that exacerbate chylomicronaemia include alcohol use, diets high in fat and simple carbohydrates, uncontrolled diabetes, obesity, insulin resistance, non-alcoholic fatty liver disease, pregnancy and medications including corticosteroids, oral oestrogens, psychotropic drugs, and immunosuppressants.^1,2^ This condition is called multifactorial chylomicronaemia syndrome (MCS), and it is the most common form of chylomicronaemia.^2^

In contrast, a tiny subset of patients with severe hypertriglyceridaemia has familial chylomicronaemia syndrome (FCS) caused by an inherited complete loss of LPL activity.^1^ FCS is found in 1 in 100,000–1,000,000 individuals.^4,5^ FCS results from biallelic pathogenic variants in the *LPL* gene encoding LPL or in the glycosylphosphatidylinositol-anchored high-density lipoprotein binding protein 1 (*GPIHBP1*), apolipoprotein A5 (*APOA5*), apolipoprotein C2 (*APOC2*) and lipase maturation factor 1 (*LMF1*) genes encoding GPIHBP1, apolipoprotein (apo) A-V, apo C-II and LMF1, respectively.^1^ Because FCS is a single-gene disorder, symptoms, including failure to thrive, can begin in infancy or childhood.^1^ FCS is stressful for patients, families and care providers since current treatment options are very limited, except for a draconian fat-restricted diet that provides around 15% of calories as fat to reduce the substrate enterocytes use to synthesize chylomicrons.^2,4^ Traditional TG-reducing treatments, such as fibrates, niacin and omega-3 fatty acids, are minimally effective.^4^

In pre-genomic times, FCS was diagnosed biochemically by demonstrating the absence of lipolytic activity in plasma after heparin infusion, although the assay was cumbersome and somewhat variable.^1,2^ Currently, genetic diagnosis is the gold standard for FCS.^1,5^ Reducing TGs to <5.6 mmol/L (<500 mg/dL) in FCS is associated with reduced pancreatitis risk.^1,4^ In adults with FCS, dietary adherence is challenging, and relapsing pancreatitis often occurs.^4^ Plasmapheresis has been suggested in some cases, but its effects are very transient; furthermore, it is invasive, incurs costs, requires specialized equipment, is not without risk and does not improve survival.^4^ Other proposed treatments, such as inhibitors of microsomal TG transfer protein, apo B, diacylglycerol acyl transferase 1 and angiopoietin-like protein 3, all have limitations.^4^ There is a critical unmet need for new treatments for FCS.^4^

Apo C-III, a 79 amino acid protein that circulates with chylomicrons and other lipoproteins, is a promising treatment target in chylomicronaemia.^6^ The effects of apo C-III on lipoprotein metabolism include promoting the secretion of TG-rich lipoproteins, interfering with LPL activity and interfering with the receptor-dependent and -independent clearance of TG-rich lipoproteins.^1^Furthermore, classical biochemical, transgenic animal, family and Mendelian randomization studies in aggregate indicate that silencing hepatic *APOC3*, for example, with antisense oligonucleotides (ASOs) or RNA interference approaches, will reduce TG levels.^1^

The value of knocking down apo C-III in FCS was first shown in the APPROACH (A study of volanesorsen [formerly IONIS-APOCIIIRx] in patients with familial chylomicronemia syndrome; ClinicalTrials.gov identifier: NCT02211209) study, a phase III, multi-institutional, randomized, double-blind, placebo-controlled trial of volanesorsen, an ASO that binds *APOC3* mRNA in the nucleus and inhibits its translation.^7,8^ In APPROACH, 66 patients were studied, 52 of whom had genetically diagnosed FCS: 33 received volanesorsen 300 mg subcutaneously once weekly, and 33 received matching doses of placebo.^7,9^ At 3 months, volanesorsen was associated with placebo-adjusted reductions of around 90% in apo C-III and TG levels, with beneficial effects found across multiple lipoprotein subfractions. However, thrombocytopenia (platelets <100,000/μL) occurred in 15 of 33 participants receiving volanesorsen compared with none who received the placebo. Two participants in the volanesorsen group had platelets <25,000/μL.^2^ Thrombocytopenia was considered to result from a chemical property of volanesorsen itself rather than from the mechanism for targeting apo C-III. The risk of thrombocytopenia necessitated routine platelet monitoring and mandated dose down-titration and/or corticosteroid administration.^2^ Volanesorsen for FCS was eventually approved with restrictions in Europe but not in North America.^10,11^

Two novel agents targeting *APOC3* are in clinical development in the hope of retaining the benefits of volanesorsen while avoiding thrombocytopenia. The first, olezarsen (formerly AKCEA-APOCIII-LRx) is an N-acetylgalactosamine (GalNAc)-conjugated ASO that enters the hepatocytes via the asialoglycoprotein receptor, which allows for reduced dosing that maintains efficacy with better safety than volanesorsen.^6,12^ In patients with moderate hypertriglyceridaemia who received subcutaneous injections of olezarsen, mean TG levels were reduced by up to 67% compared with placebo.^3^ Occasional mild injection-site reactions but no thrombocytopenia were noted.^3^ An on-going phase III, multi-centre, randomized, double-blind, placebo-controlled study called BALANCE (A study of olezarsen [formerly known as AKCEA-APOCIII-LRx] administered to patients with familial chylomicronemia syndrome; ClinicalTrials.gov identifier: NCT04568434) is evaluating the efficacy of olezarsen administered monthly over a year via subcutaneous injection in adults with FCS.^13^

A second novel apo C-III antagonist, ARO-APOC3, is a GalNAc-bound RNA interference that targets *APOC3* mRNA within the cytoplasm of hepatocytes, resulting in prolonged suppression of apo C-III.^6,14^ A phase I trial in patients with severe hypertriglyceridaemia, including some with FCS, showed that ARO-APOC3 was associated with reductions of up to 96% and 92% in apo C-III and TG levels, respectively.^14^ An ongoing phase III, randomized, double-blind, placebo-controlled trial called PALISADE (AROAPOC3-3001; Study of ARO-APOC3 in adults with familial chylomicronemia syndrome; ClinicalTrials.gov identifier: NCT05089084) commenced in January 2022 to evaluate the efficacy and safety of ARO-APOC3 in patients with FCS.^15^ A planned 60 adults with TG ≥10 mmol/L (≥880 mg/dL) and genetically diagnosed FCS are being randomized to receive either ARO-APOC3 25 mg or 50 mg subcutaneously every 3 months (n=20 for each dose) or volume-matched saline placebo (N=10 for each treatment group).^15,16^ The primary endpoint is the change from the baseline of fasting TG at 10 months, with several secondary and exploratory endpoints.^15^ After 12 months, the participants will be invited to continue in an open-label extension study.^16^

Both the PALISADE and BALANCE studies offer new hope for patients with FCS, who are among the most challenging in lipidology to manage. Achieving sustained TG reduction alongside other potential metabolic and clinical benefits and minimal adverse effects would be a major advance. Furthermore, a favourable benefit-to-risk ratio of new treatments in FCS could have implications for patients with MCS, which is at least 50- to 100-times more common than FCS.^1^ While the relative risk of pancreatitis is lower in patients with MCS than in those with FCS, the absolute number of acute pancreatitis episodes across all MCS patients is substantial.^17^ A clinically relevant subgroup (perhaps 5–10%) of patients with MCS with refractory or sustained chylomicronaemia might particularly benefit from ARO-APOC3 or olezarsen. Such patients are operationally equivalent to those with genetic FCS. New nomenclatures such as ‘refractory’ or ‘sustained’ chylomicronaemia might be appropriate for patients with true genetic FCS; extreme cases of MCS due to heterozygous pathogenic variants or high polygenic risk of hypertriglyceridaemia^18^; other unusual combinations of rare genetic variants; low LPL biochemical activity with high clinical suspicion for FCS based on clinical scoring^19^ but negative for genetic findings; persistent autoantibodies against GPIHBP1 or LPL^20^; or potential new non-Mendelian disease mechanisms, such as methylation. While most patients with chylomicronaemia do not have true genetic FCS, some might still similarly benefit from effective and well-tolerated anti-*APOC3* therapies. Future clinical trials comparing anti-*APOC3* therapies with other newer agents will decide their role not only in patients with FCS but also in the more common MCS and other forms of hypertriglyceridaemia.
